# A treadmill training program in a gamified virtual reality environment combined with transcranial direct current stimulation in Parkinson’s Disease: Preliminary results of a mixed methods randomized controlled trial

**DOI:** 10.1007/s10072-026-09136-8

**Published:** 2026-06-12

**Authors:** Pere Bosch-Barceló, Carlos Tersa-Miralles, Oriol Martínez-Navarro, Maria Masbernat-Almenara, Anni Pakarinen, Helena Fernández-Lago

**Affiliations:** 1 Department of Nursing and Physiotherapy, University of Lleida, Lleida, Spain; 2 Research Group of Health Care (GReCS), Lleida Institute for Biomedical Research Dr. Pifarré Foundation (IRBLleida), Lleida, Spain; 3 Lleida Institute for Biomedical Research Dr Pifarré Foundation, IRBLleida, Lleida, Spain; 4 Department of Nursing Science, University of Turku, Turku, Finland; 5 Consolidated Research Group: Society, Health, Education and Culture (GESEC), University of Lleida, Lleida, Spain

**Keywords:** Parkinson’s Disease, Gait, Virtual Reality, Transcranial Direct Current Stimulation, Motor Activity

## Abstract

**Background:**

Gait impairments in Parkinson’s disease (PD) worsen under cognitive load and often persist despite medication. Combining treadmill training with gamified virtual reality environments (GVRE) and transcranial direct current stimulation (tDCS) may address both motor and cognitive contributors. This study evaluated the effects and experiences of a cognitive-motor gait training program integrating GVRE and tDCS.

**Methods:**

In this mixed-methods RCT, 23 participants with mild-to-moderate PD were randomized to: (1) treadmill (*n* = 8), (2) treadmill+GVRE (*n* = 8), or (3) treadmill+GVRE+tDCS (*n* = 7). Participants completed 12 sessions over six weeks. Primary outcomes included spatiotemporal gait parameters and executive function under single- and dual-task conditions. Secondary outcomes included balance, motor severity, fear of falling and quality of life. Interviews explored perceived effects and motivational factors.

**Results:**

The intervention was safe and well tolerated, with high attendance (92%). No statistically or clinically meaningful changes were observed in gait speed. Balance improved across groups (MiniBESTest + 1.48 points; *p* = 0.0003). A timepoint-specific improvement in cadence during motor dual-task walking was observed in the treadmill+GVRE+tDCS group compared with treadmill. Interviews revealed increased walking confidence and adoption of mobility strategies in complex walking situations.

**Conclusions:**

This preliminary mixed-methods RCT supports the feasibility and acceptability of combining treadmill training with GVRE and tDCS in people with mild-to-moderate PD. While the intervention did not yield clinically meaningful improvements in gait speed, exploratory outcomes offered insight into participant experiences. Reported gains in confidence, self-monitoring, and walking strategies provide useful context. Findings should be interpreted cautiously. Larger, adequately powered, sham-controlled trials are needed to determine efficacy.

**Supplementary Information:**

The online version contains supplementary material available at 10.1007/s10072-026-09136-8.

## Introduction

Parkinson’s disease (PD) is the fastest-growing neurological condition globally, with its prevalence projected to exceed 12 million cases by 2040 [1, 2]. Characterized by cardinal motor symptoms such as bradykinesia, rigidity, rest tremor and postural instability, PD also presents with a range of non-motor impairments, including progressive deficits in executive function, attention, and dual-tasking capacity [3]. These cognitive-motor interactions are especially critical for safe mobility: walking while talking, navigating unpredictable environments, or responding to complex stimuli all require dynamic integration of attentional and motor resources. In people with Parkinson’s disease (PwPD), such dual-task (DT) conditions often lead to slower gait, increased variability, and reduced stability, which are key predictors of falls, loss of independence, and diminished quality of life [4, 5].

Pharmacological treatments offer only partial relief. While dopaminergic therapies improve motor symptoms under single-task conditions, their effect on cognitive-motor interference is limited and, in some cases, paradoxical [6]. As such, non-pharmacological approaches have gained increasing relevance in addressing real-world mobility challenges. Among these, treadmill-based gait training has consistently demonstrated improvements in spatiotemporal gait parameters and motor control in PwPD [7]. Yet traditional treadmill training may fall short in preparing patients for cognitively demanding walking scenarios. This has prompted the development of dual-task training paradigms that embed cognitive stimuli into gait exercises, with promising results for both motor and cognitive outcomes [8].

Virtual Reality (VR) has emerged as a particularly compelling medium for such training. By simulating rich, immersive, and ecologically valid environments, VR enables the controlled interaction with obstacles, distractors, and executive demands during gait while preserving safety and repeatability. Landmark studies, such as the V-TIME trial, have shown that integrating non-immersive VR with treadmill training can significantly reduce fall risk in older adults, including those with PD [9]. However, the field has since struggled to sustain conceptual or technical advancement. Much of the VR-based literature in PD continues to rely on commercial gaming platforms (e.g., Wii Fit, Kinect Adventures), which are not designed for populations with slowed movement, rigidity, or tremor [10–12]. These systems often lack sufficient adaptability, clinical relevance, or progression mechanisms for meaningful neurorehabilitation.

One underutilized dimension in this context is gamification. Distinct from VR per se, gamification refers to the integration of game mechanics such as feedback mechanisms, level progression, performance scoring, or narrative elements into non-game contexts to enhance motivation, engagement, and learning. Gamification holds particular promise for older adults and neurological populations by increasing adherence, encouraging cognitive investment, and potentially reinforcing plasticity-driven learning [13, 14]. Despite its theoretical relevance, few controlled studies have examined whether gamified virtual environments offer tangible advantages over traditional or non-gamified VR in PD rehabilitation.

Compared to healthy individuals, PwPD exhibit impairments in motor skill consolidation and automatization. To compensate, they tend to engage extra brain areas and modify the patterns of effective neural connectivity [15]. These complications may limit the effectiveness of traditional physical therapy, so there is a growing interest in neuromodulatory techniques that can facilitate better re-learning of motor patterns. Anodal transcranial direct current stimulation (tDCS), which increases excitability in the stimulated cortical region, has shown potential to enhance working memory, executive function, and motor acquisition when applied over the left dorsolateral prefrontal cortex (DLPFC), a key region involved in attentional allocation, error monitoring, and Dual Task (DT) coordination [16, 17].

In PD, early-phase studies combining tDCS with physical or cognitive tasks have shown mixed but encouraging results across a range of outcomes including gait, bradykinesia, cognitive flexibility, and fatigue. Variability in findings may reflect differences in electrode placement, current intensity, disease stage, and concurrent task demands [18, 19]. Nevertheless, tDCS remains a promising adjunct due to its safety, portability, and potential to be deployed alongside scalable rehabilitation platforms. In the context of cognitively demanding gait training, tDCS over DLPFC may enhance attentional focus and adaptability during complex movement tasks. This hypothesis remains largely untested in trials integrating simultaneous cognitive-motor stimulation and gamified VR environments, a gap this study seeks to address.

The aim of this three-arm mixed methods RCT is to evaluate the effects of the inclusion of a GVRE to a 6-week treadmill training program with anodal-tDCS in PWPD on gait parameters during Single and DT, clinical outcomes and executive functions as well as participant experience and motivation; in comparison to a GVRE treadmill training program; and compared to a treadmill training as a control.

## Materials and methods

### Trial design

**Fig. 1:**
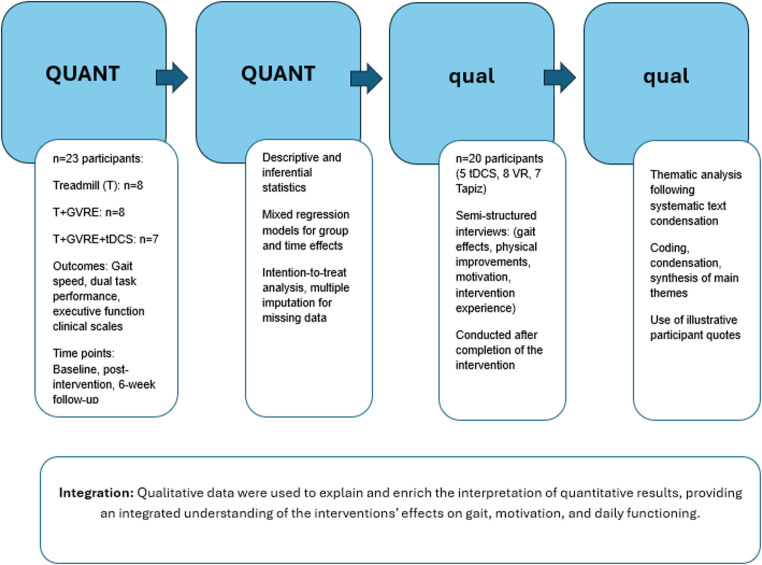
Explanatory sequential mixed methods design flowchart, adapted from Tovin & Wormley [19]

This study followed a mixed methods explanatory sequential design [20], beginning with a single-blind, single-center, three-arm, prospective Randomized Controlled Trial (RCT) as the quantitative component, followed by a qualitative phase to further explore and explain the trial findings, as detailed in Fig. 1 [21]. This study was conducted in Lleida, Spain, at the Lleida Institute for Biomedical Research Dr. Pifarré Foundation (IRBLleida). Eligible participants were randomly distributed in three groups: (1) Treadmill; (2) Treadmill+Gamified Virtual Reality Environment (GVRE); (3) Treadmill+GVRE+tDCS. Detailed information on quantitative assessments can be found in the published study protocol for this RCT [22].

### Participants

A total of 23 participants were recruited, mainly through the local PD association (Associació de Parkinson de les Terres de Lleida), distribution of fliers and contact with local news media.

The following inclusion criteria were applied:

- Aged 50 or older
- Idiopathic PD
- Stage II – III in the Hoehn & Yahr (H&Y) scale during ON state
- Ability to walk for 10 minutes independently without stopping

The exclusion criteria were the following:

- Significant cognitive decline based on mini mental status examination (MMSE <23)
- Severe auditory or visual deficits
- Other neurological/psychiatric conditions
- Any kind of cardiovascular complications that contraindicate physical activity
- Clinical history of any brain surgery or deep brain stimulation device

Subjects that changed their medication while participating in the RCT were considered as non-retention subjects.

### Ethics approval

The study received approval from the Comitè d’Ètica d’Investigació amb Medicaments (CEIM) – Hospital Universitari Arnau de Vilanova (reference CEIC-2231). All participants provided written informed consent prior to enrollment and were fully briefed on the study procedures by a member of the research team.

### Randomization and blinding

Randomization and allocation concealment were carried out using a computer-generated random number technique. Block randomization with a block size of three was applied, stratified by two variables: age and disease severity. Participants were assigned to groups in a 1:1:1 ratio. The randomization list was created using Study Randomizer software by a postdoctoral researcher who was blinded to the study and not otherwise involved. To ensure allocation concealment, opaque sealed envelopes were prepared and only opened at the time of assignment in front of the participants. tDCS was not sham-controlled; participants and researchers were aware of whether stimulation was delivered. To mitigate expectation and performance bias, outcome assessments were conducted by an evaluator blinded to group allocation, and standardized instructions were used across groups.

### Interventions

The intervention spanned six weeks, comprising two sessions per week for a total of twelve sessions. This training framework was adapted from protocols established by Mirelman et al. [9] and Shulman et al. [23] and further modified to suit the specific features of the GVRE setup and training demands based on feedback from a Feasibility trial testing the intervention structure [24]. Comprehensive details regarding the intervention structure, GVRE system, tDCS application, and session duration are provided in the published protocol [22].

In the GVRE arms, treadmill walking was combined with a gamified virtual environment designed to reproduce cognitively demanding walking situations in a controlled and safe setting. The virtual task participants faced was a “walk your dog” situation, which included goal-directed walking challenges, visual stimuli, obstacle-related demands, and attention-demanding elements intended to promote cognitive-motor engagement during gait. Task difficulty was progressed individually across sessions by adapting treadmill speed, distraction and obstacle frequency, visual cluttering and limited visibility according to participant tolerance, safety, and performance. Feedback was provided through the GVRE through performance-related cues and overall scores after completing each session. The aim was to maintain training at a moderate perceived intensity while preserving safe gait execution. In the Treadmill+GVRE+tDCS arm, anodal tDCS was applied with the anode on the left dorsolateral prefrontal cortex (DLPFC) and the cathode on the right DLPFC, concurrently with the GVRE treadmill training as an adjunct intended to modulate cognitive-motor control processes during the cognitively demanding gait practice. A detailed summary of the treadmill, GVRE and tDCS components structure, progression, feedback, and procedures is provided in Supplementary Table S1.

During each session, participants were secured using a harness attached to a fixed safety frame to prevent falls, though no body weight support was provided. Sessions were supervised by a licensed physiotherapist with expertise in Parkinson’s disease, neurorehabilitation, and tDCS. Exercise intensity was consistently monitored to stay within moderate levels; if a participant exceeded this threshold, the activity was paused immediately, and a rest period was introduced.

### Outcome measures

#### Quantitative measures

The primary outcome measure was gait speed, evaluated both during the Intermittent Shuttle Walking Test and under DT conditions. Additional gait parameters were treated as secondary outcome measures. All assessments were collected at baseline (T0), post-intervention (T1), and follow-up (T2) and at approximately the same time of day for each participant across visits to reduce diurnal variability. Participants were assessed in their usual ON-medication state.

#### Gait parameters: intermittent shuttle walking test

Participants completed ten round trips along an 8-meter walkway at five distinct walking speeds: preferred, slow, very slow, fast, and very fast (10 × 5 × 8 m). This protocol, adapted from Ambrus et al. (25), provided a reliable basis for establishing a linear relationship between stride length and walking speed. Gait was monitored using the APDM^®^ inertial sensor system (Opal, APDM, Portland, OR, USA), capturing the following parameters: gait speed (m/s), stride length (cm) and cadence (Hz). The stride length–cadence relationship (SLCadRel) was also calculated as an index of gait scaling across speeds, derived from the linear association between stride length and cadence across the five speed conditions [25].

#### Gait parameters: dual task conditions

Participants were also instructed to walk along the same 8-meter aisle while performing a concurrent task. Three types of DT conditions were assessed:

- Arithmetic DT (DTA): Participants walked while subtracting 7 sequentially from different numbers (e.g. 100).
- Verbal Fluency DT (DTV): Participants walked while naming words that started with a given letter.
- Motor DT (DTM): Participants walked while holding a tray with a half-filled glass of water using both hands.

The mental tracking and verbal fluency tasks were used to evaluate attention during walking. Participants were not given specific instructions on whether to prioritize walking or the secondary task. To control for learning or fatigue effects, the order of DT conditions was randomized. Each task was performed three times, with a brief rest between trials. DT costs were calculated using the formula proposed by Plummer & Eskes [26] to assess the effects on performance of each situation. Average values from the second and third trials were used for data analysis.

### Secondary outcomes and descriptors

#### Executive function testing

Executive functioning was evaluated using two tasks administered via the Psychology Experiment Building Language (PEBL2) [27], an open-source platform available at http://pebl.sf.net. These tests aimed to assess cognitive domains such as inhibitory control, reaction time, attention, and interference management, all relevant to the GVRE experience:

- Victoria Stroop Test: This test assessed the ability to inhibit cognitive interference. Participants completed three blocks containing color-word stimuli, and outcomes included completion time, number of responses, and interference scores [28].
- Go/No-Go Test: This task measured attention and response inhibition. Participants responded to 80 stimuli twice, with a break in the middle, for a total of 160 stimuli, of which 20% were distractors meant to be ignored. Performance was scored based on overall accuracy [29].

#### Clinical outcome measures

Several validated clinical tools were employed to assess motor symptoms, functional autonomy, and quality of life:

- Unified Parkinson’s Disease Rating Scale – Part III (UPDRS-III): This scale was used to measure motor function impairments specific to Parkinson’s disease [30].
- Hoehn and Yahr Scale (H&Y): This staging tool was applied to classify disease severity, focusing on unilateral versus bilateral symptoms and postural reflex impairment [31].
- Parkinson’s Disease Questionnaire-39 (PDQ-39): A 39-item self-report tool that evaluated disease-specific health-related quality of life [32].
- Mini-BESTest: A 14-item test used to assess dynamic balance, covering anticipatory and reactive postural adjustments, sensory orientation, and gait-related balance control [33].
- Falls Efficacy Scale – International (FES-I): This 16-item questionnaire evaluated participants’ concern about falling during various daily activities [34].
- Montreal Cognitive Assessment (MoCA): This 30-point test provided a broad overview of cognitive function, including memory, executive function, visuospatial skills, language, abstraction, and orientation [35].

#### Qualitative measures

As participants completed their intervention programs, individual semi-structured interviews were conducted to explore their perceptions and experiences in relation to observed changes (or lack of change) in quantitative outcomes and the real-world relevance of the intervention. Of the 23 participants enrolled, 20 were interviewed; three could not participate due to varying reasons. The interview guide focused on participants’ perceived changes in gait and mobility, both in routine and challenging environments (e.g., walking while multitasking or navigating obstacles), as well as the impact of the intervention on daily activities.

Additional themes included factors influencing adherence to the program and the evolution of participants’ motivation over time. Interviewers also explored what aspects of the training were most engaging or discouraging, and how these influenced participants’ willingness to continue physical activity beyond the study.

All interviews were conducted in a private setting by trained research staff, recorded with consent, and transcribed verbatim. The goal was to gain insight into the real-world relevance of the intervention and to identify facilitators or barriers to sustained engagement in mobility-related rehabilitation.

## Analysis

### Integration of quantitative and qualitative data

Quantitative and qualitative data were analyzed separately and integrated within an explanatory sequential mixed methods framework. Quantitative outcomes were analyzed first, and the subsequent qualitative interviews were used to explain, contextualize, and elaborate the quantitative results. Integration occurred at the interpretation and reporting stage using a narrative weaving technique and a joint display [36] that aligned quantitative outcomes with qualitative themes within four Results domains (Changes in Gait Parameters Under Single and DT Conditions; Cognitive-Motor Integration and DT Effects; Clinical Outcomes; Engagement, Motivation, and Acceptability). Mixed-methods meta-inferences were developed using three pathways: confirmation (convergent evidence), expansion (complementary insights), and discrepancy (divergent findings requiring contextual interpretation).

### Quantitative analysis

All statistical analyses were conducted following a blinded intention-to-treat approach. The significance level was set at α = 0.05. Descriptive statistics were used to summarize the data: categorical variables were reported as frequencies and percentages, while continuous variables were described using means and standard deviations or medians with interquartile ranges, depending on the distribution.

Baseline sociodemographic and clinical characteristics were examined across groups using descriptive comparisons. Data normality and homogeneity of variances were assessed using the Kolmogorov-Smirnov and Levene’s tests, respectively. Although Freezing of Gait (FoG) was initially considered a potential confounding variable in the analysis plan, it was not included in the final statistical models, as no participants in the sample exhibited clinically significant FoG. This aligns with recent concerns in the literature regarding the limitations of current FoG assessment methods in both clinical and research settings. For instance, Gilat et al. [37] have noted that commonly used FoG questionnaires and observational tools may lack sensitivity, context specificity, or ecological validity, particularly when applied outside of triggering environments or in individuals with mild to moderate PD.

Group differences were analyzed using a mixed-effects regression model, incorporating both between-subjects factors (Treadmill, Treadmill+GVRE, Treadmill+GVRE+tDCS) and within-subjects factors (baseline, post-intervention (T1), and six-week follow-up (T2)). For variables with non-normal distributions, log transformation was applied prior to analysis. Missing data were handled using multiple imputation via chained equations [38].

Results were reported as estimated differences with corresponding 95% confidence intervals. Changes in gait speed were also interpreted in relation to their Minimal Clinically Important Difference (MCID) in accordance with criteria established by Hass et al. [39].

### Qualitative analysis

A thematic analysis [40] was conducted to explore participants’ subjective experiences with the intervention, particularly related to gait, mobility in everyday and complex environments, and motivational factors affecting adherence. All interviews were transcribed verbatim and analyzed using an inductive coding approach. Three researchers (CTM, HFL & PBB) familiarized themselves with the full data set through repeated listening and reading before conducting line-by-line coding. To support data management, initial codes were provisionally grouped using broad content areas reflected in the interview guide; however, code refinement and theme development were driven by patterns emerging from the data.

Themes were developed to capture participants’ perceptions of changes in walking ability and challenges in multitasking during gait, confidence in daily mobility, evolution of clinical outcomes and the role of motivation and engagement in sustaining participation. Coding and thematic development were reviewed collaboratively by two researchers (CTM & PBB), and discrepancies were resolved through discussion to ensure analytic rigor. After qualitative themes were finalized, they were aligned with the four Results domains used for integrated reporting to facilitate side-by-side interpretation with quantitative findings. Representative quotations were selected to illustrate each theme and to support interpretation of how participants’ experiences related to the quantitative outcomes.

## Results

Detailed demographic data can be found in Table 1. In line with the explanatory sequential design, qualitative findings are presented to help explain and contextualize the quantitative results, with integrated interpretation summarized in the joint display (Table 2).

**Table 1:** Participant characteristics at baseline (T0)

	T (*n* = 8)	T+GVRE (*n* = 8)	T+GVRE+tDCS (*n* = 7)	Total (*n* = 23)
Age, years (mean ± SD)	70.2 ± 4.5	68.0 ± 8.6	67.3 ± 13.0	68.6 ± 8.8
Male, n (%)	6 (75%)	6 (75%)	5 (71%)	17 (74%)
Most affected side (right), n (%)	6 (75%)	4 (50%)	4 (57%)	14 (61%)
Years since diagnosis, median [IQR]	8.0 [4.8–10.0]	4.5 [2.8–8.3]	6.0 [3.5–7.0]	6.0 [3.5–8.5]
MMSE (mean ± SD)	28.5 ± 1.2	28.6 ± 1.6	28.4 ± 1.8	28.5 ± 1.5
BDI (mean ± SD)	9.2 ± 5.2	7.4 ± 6.0	7.1 ± 4.1	8.0 ± 5.0
IPAQ (low / moderate / high), n (%)	1 (12%) / 5 (62%) / 2 (25%)	0 (0%) / 4 (50%) / 4 (50%)	1 (14%) / 4 (57%) / 2 (29%)	2 (9%) / 13 (57%) / 8 (35%)
MoCA (baseline), mean ± SD	23.5 ± 4.8	24.6 ± 1.4	24.1 ± 6.1	24.1 ± 4.3
Hoehn & Yahr stage (2 / 3), n (%)	6 (75%) / 2 (25%)	6 (75%) / 2 (25%)	6 (86%) / 1 (14%)	18 (78%) / 5 (22%)
MDS-UPDRS III (baseline), mean ± SD	36.8 ± 14.8	40.4 ± 9.1	30.0 ± 6.2	36.0 ± 11.2

Abbreviations: *BDI*, Beck Depression Inventory; *GVRE*, gamified virtual reality environment; *IPAQ*, International Physical Activity Questionnaire; *IQR*, interquartile range; *MDS-UPDRS III*, Movement Disorder Society–Unified Parkinson’s Disease Rating Scale, Part III (motor examination); *MMSE*, Mini-Mental State Examination; *MoCA*, Montreal Cognitive Assessment; *PwPD*, people with Parkinson’s disease; *SD*, standard deviation; *T*, treadmill-only; *tDCS*, transcranial direct current stimulation

**Table 2:** Joint display of integrated quantitative and qualitative findings

Results category	Quantitative results	Qualitative results	Integration pathway	Mixed-methods meta-inference
1) Changes in Gait Parameters Under Single and DT Conditions	GS ↔ (no meaningful change; below MCID) CAD Δ (DTM cadence ↓ in T+GVRE+tDCS at T1 vs. T; DTV cadence ↑ overall) SLCad relationship SLOPE Δ/↑ (T+GVRE+tDCS ↑ at T1) SLCad relationship INT Δ (T+GVRE+tDCS ↓ at T1; T+GVRE ↑ at T2).	Participants frequently reported perceived walking improvements (confidence, steadiness, stride), but with heterogeneity (some no change/worse; some ceiling effects due to already high activity). Quote: “Because I’m more relaxed, I’m more secure; because I’m more secure, I take strides with more confidence.” (08, T+GVRE+tDCS)	Discrepancy + Expansion	Objective mean gait speed did not shift, but participants often described meaningful gains in confidence and stride strategy; the regulation metrics (slope/intercept) provide partial mechanistic support that change may occur in control/strategy rather than speed.
2) Cognitive-Motor Integration and DT Effects	DTcost ↔ GNG Δ/↑ (T+GVRE+tDCS improved at T2) Stroop Δ (temporary worsening in T at T1, not persistent at T2)	Participants described improved vigilance and situational management in complex environments (obstacles, attention allocation), while others reported no benefit due to baseline competence in multitasking. Quote: “It made me observe myself when walking… lifting my foot when I saw the obstacle.” (06, T+GVRE)	Discrepancy + Expansion	Standard dual-task gait metrics remained stable, yet participants reported context-specific “real-world” cognitive-motor adaptations; a delayed inhibitory-control signal supports a plausible cognitive-control pathway not captured by DT gait averages.
3) Clinical Outcomes	UPDRS Δ (near clinically meaningful improvement at T1 in GVRE arms; T improves at T2; T+GVRE+tDCS worsens from T1 to T2) MiniBEST ↑ (overall improvement at T1 and sustained at T2, modest change).	Participants framed benefits as improved well-being/fitness and, importantly, maintenance/prevention of decline. Quote: “If I hadn’t done this treatment, I don’t know how I’d be now. Maybe I’d be worse, but I don’t know.” (05, T+GVRE+tDCS)	Confirm + Expand	Quantitative balance improvement aligns with perceived functional stability; qualitative discourse strengthens clinical relevance by emphasizing maintenance as a meaningful outcome in PD even when MCID thresholds are not met.
4) Engagement, Motivation, and Acceptability	Feasibility indicators: high attendance, high compliance and no adverse events	Strong acceptability themes: supportive atmosphere and supervision; novelty and discovery driving motivation in GVRE; treadmill-only described as monotonous; motivation evolved over time and influenced intentions to continue physical activity. Quote: “What kept me motivated was the expectation of discovering something new each day, what we would do, what I’d see, the landscape, the challenge.” (07, T+GVRE) “Just doing the treadmill, just walking, without anything else. It was not very stimulating, I think. Well, that’s the impression I have.” (15, T)	Confirm + Expand	Quantitative feasibility is corroborated and explained by qualitative mechanisms: novelty, engagement, and relational support highlighting GVRE’s value as an adherence/implementation enhancer even when primary clinical effects are modest.

Legend for quantitative cells: ↔ = no statistically/clinically meaningful change; ↑ = significant improvement/increase; ↓ = significant decrease; Δ = mixed / timepoint-specific / arm-specific effect; — = not applicable to theme. Abbreviations: PD, Parkinson’s disease; T, treadmill-only; GVRE, gamified virtual reality environment; tDCS, transcranial direct current stimulation; GS, gait speed; CAD, cadence; DT, dual task; DTM, motor dual-task condition; DTV, verbal fluency dual-task condition; DTcost, dual-task cost; SLCad, stride length–cadence relationship; SLOPE, slope of the stride length–cadence relationship; INT, intercept of the stride length–cadence relationship; GNG, Go/No-Go task; MDS-UPDRS III, Unified Parkinson’s Disease Rating Scale, Part III; MCID, minimal clinically important difference

### Participant characteristics

Twenty-three participants joined the intervention, with a mean age of 68.6 years (SD = 8.83), and 73.9% were male. The majority (60.9%) had the right side of the body most affected by PD, and the mean time since diagnosis was approximately 6.5 years. Cognitive function, assessed by the MMSE (mean = 28.5) and MoCA (mean = 24.1), indicated relatively preserved cognition. Baseline depression symptoms (Beck Depression Inventory score = 7.96) were low. Most participants reported moderate (56.5%) or high (34.8%) physical activity levels based on the International Physical Activity Questionnaire (IPAQ). Disease severity was moderate, with 78.3% in H&Y stage II and 21.7% in stage III. No significant differences were found between the intervention arms in these baseline characteristics.

### Changes in gait parameters under single and dual task conditions

At baseline, gait speed under single-task conditions averaged 1.00 m/s (SD = 0.20), with expected decrements under DT conditions: DTM (mean = 0.92 m/s), DTV (mean=0.83m/s), and DTA (mean=0.81m/s). Linear mixed models revealed no statistically significant group-level changes in gait speed at either T1 or T2. Estimated changes across all groups remained below the minimal clinically important difference (MCID = 0.22 m/s), with confidence intervals for all comparisons overlapping zero.

Cadence at baseline averaged 110 steps/min (SD = 11.1), with declines under DT conditions: DTM (mean = 108), DTV (mean = 100), and DTA (mean = 99.7). A significant reduction in DTM cadence at T1 was observed in the Treadmill+GVRE+tDCS group compared to Treadmill (−7.00 steps/min; 95% CI: −13.50 to − 0.50; *p* = 0.036). Across all intervention arms and both T1 and T2, a significant overall increase in cadence was detected during the DTV condition (mean change + 1.90 steps/min; 95% CI: 0.02 to 3.75; *p* = 0.0492). No significant differences were found between changes at T1 and T2.

Stride length under single-task conditions averaged 1.08 m (SD = 0.17), with expected DT reductions: DTM (mean = 1.02 m), DTV (mean = 0.98 m), DTA (mean = 0.97 m). No group × visit interactions were statistically significant overall. However, in the Treadmill+GVRE+tDCS group, a small but significant increase in the stride length–cadence relationship slope was detected at T1 (adjusted difference + 0.002; 95% CI: 0.000 to 0.004; *p* = 0.048), corresponding to a standardized effect of ≈ 0.62 SD.

At baseline, the mean stride length–cadence intercept (an indicator of baseline stride length for a given cadence) was − 0.08 (SD = 0.34), with no significant group differences (*p* = 0.11). A significant visit × intervention interaction was found (*p* = 0.044), suggesting different group trajectories. At T1, the Treadmill+GVRE+tDCS group showed a reduction in intercept (adjusted difference − 0.33; 95% CI: −0.65 to − 0.02; *p* = 0.039), suggesting a lower stride offset relative to cadence (i.e., a shorter-than-expected stride at a given stepping rate). At T2, the Treadmill+GVRE group showed a significant increase in intercept (adjusted difference + 0.41; 95% CI: 0.07 to 0.74; *p* = 0.020). These changes, estimated around 0.3–0.4 units, correspond to ~ 0.8–1 SD.

This pattern of no significant group-level change, limited intervention effects, and sub-MCID changes was consistent across DTM, DTV, and DTA conditions for all gait variables.

In contrast with the limited objective improvements in gait parameters, several participants across intervention arms described experiencing positive changes in their mobility, particularly in terms of walking confidence, stride regulation, and perceived physical fitness. These reported effects were often framed as gradual shifts in awareness, self-regulation, and motor strategy that extended beyond the training sessions themselves:

“Because I’m more relaxed, I’m more secure; because I’m more secure, I take strides with more confidence.” (08, T+GVRE+tDCS)

“I go out walking for an hour every day, and maybe before I used to take shorter steps, and now I try to make them longer, because I move forward more and get less tired. I mean, that’s something I discovered with the machine (treadmill); the machine helps you lengthen your stride. And so now, when I notice it… I try to take longer steps to move more and get less tired.” (13, T)

Some of the participants directly pointed at the intervention not having an impact on their gait due to their current physical condition and training experience, believing it more useful for sedentary participants:

“So, what is clear with this type of program is that people who come to the program and don’t usually do any kind of exercise, like walking or things like that, I think they’ll probably realize that after completing the program, it’s easier for them.” (07, T+GVRE)

“Well, I think that for people who don’t walk or exercise, walking every day, well, it might be motivating for them. But the thing is, I already exercise.” (05, T+GVRE+tDCS)

A recurring theme was the perception that the intervention period was too brief to produce meaningful or lasting effects on gait. Participants expressed uncertainty about whether the program had been truly beneficial, noting that the short timeframe limited their ability to evaluate its impact.

“There are. improvements or not, you’re the ones who will look into that… But the perception one has, given the short duration and time frame in which you’re applying it, is that it doesn’t really give you much room to know whether it’s actually been beneficial, whether it’s made a difference or not.” (08, T+tDCS)

For some participants, especially among the Treadmill group, the intervention was not seen as beneficial for gait, with perceptions of the training protocol not being useful for PD, or even feeling worse than at the beginning:

“I don’t know whether the study was enough or not. I don’t know, but honestly, I think I did my part, I followed the program as instructed, and I was going fast, but now when I walk in the street and so on, well… I don’t notice any difference.” (14, T)

“No. I’ve noticed I’m getting worse.” (04, T)

### Cognitive-motor integration and dual task effects

#### Quantitative findings

At baseline, DT effects on gait speed, cadence, and stride length showed expected DT costs across all groups. However, no statistically or clinically significant intervention effects were observed at T1 or T2 for any DT effect on gait variables under DT conditions.

For executive function testing, group-level analysis revealed no significant differences in overall Stroop task performance across conditions. However, a model-estimated significant median increase in the time to complete the Stroop Part W was detected at T1 for the Treadmill+GVRE group (*p* < 0.05), potentially indicating a temporary worsening in word condition response time. This effect did not persist at the follow-up visit.

Baseline accuracy in the Go/No-Go task was high and similar across all groups (mean ≈ 0.91, SD ≈ 0.07). No significant group-level differences were observed at either T1 or T2. Nonetheless, a model-estimated significant increase in accuracy was observed in the Treadmill+GVRE+tDCS group at T2 (*p* < 0.05)

In contrast with the lack of quantitative results, participants reported increased awareness of movement and developed more deliberate walking strategies, particularly in response to cognitive challenges:

“It made me observe myself when walking… lifting my foot when I saw the obstacle.” (06, T+GVRE)

“I’m more responsible for how I walk… Now I get up slowly, check for anything on the ground…” (10, T)

However, reports on actual improvements in multitasking abilities were mixed, with perceptions from participants ranging from useful in urban environments to no improvements for their daily routines:

“Well, maybe. For example, when you’re walking down the sidewalk, you come across things like Telefónica manhole covers. And through the way you learn to walk with obstacles, you adjust your step just enough to avoid and not step on a manhole cover that’s in the way.” (11, T+GVRE+tDCS)

“I don’t know if it has had any influence or not. What I do know is that I don’t have any problem walking and talking, because I walk every morning and I don’t go alone, three of us go walking together, and we talk the whole time.” (13, T)

### Clinical outcomes

At baseline, mean UPDRS-III scores were 36.0 (SD = 11.2), with no significant differences between groups (*p* = 0.20). At T1, both Treadmill+GVRE (− 5.18 points; *p* = 0.093) and Treadmill+GVRE+tDCS (− 6.27 points; *p* = 0.067) showed improvements near or above the 5-point mark, though not statistically significant. At T2, Treadmill+GVRE maintained this benefit, while Treadmill+GVRE+tDCS showed significant deterioration from T1 (+ 2.77 points; *p* = 0.005). The Treadmill group improved gradually, reaching statistical significance at T2 (− 4.77 points; *p* = 0.015).

At baseline, the mean MiniBESTest score was 22.5 (SD = 3.82), with no significant differences between intervention groups. When analyzing data across all groups and visits, there was a statistically significant overall improvement in balance performance following the interventions. The model-estimated average change from baseline was + 1.32 points at T1 (95% CI: 0.59 to 2.05) and + 1.72 points at T2 (95% CI: 0.94 to 2.50). This represents an overall predicted mean improvement of + 1.48 points (95% CI: 0.80 to 2.16; *p* = 0.0003). No significant difference was found between the amount of improvement at T1 versus T2 (LRT *p* = 0.225), suggesting that balance improvements achieved through the interventions were maintained over time. No significant changes were observed for quality of life and fear of falling.

Participants expressed a wide spectrum of perceptions regarding the intervention’s overall impact on their health and functioning. For many, particularly those who were less physically active prior to the study, the program offered a structured opportunity to reintroduce regular movement and foster a sense of progress. These participants described improvements in walking frequency, physical strength, and general well-being:

“I walk better. I walk more. I’ve gained more strength.” (01, T+GVRE)

“Well, after doing this intervention, I’ve spent all these months, let’s say… I think we did it in May. (…) In May, and then practically until the end of the year, I’ve felt… I’ve felt in very good shape. Really good shape. I guess it was this mobility thing.” (03, Treadmill+GVRE)

“If I control the speed and intensity, I last longer and stay calmer, and the medication effect lasts longer.” (05, T+GVRE+tDCS)

In contrast, participants who were already physically active or had lived with Parkinson’s for a longer time often reported little to no perceived benefit. It was stated that their pre-existing routines already incorporated similar types of activity, leading them to view the intervention as redundant or insufficiently tailored.

“I already do a lot of treadmill and exercise, but at an individual level I didn’t find an improvement.” (09, T+GVRE)

Notably, among those who did perceive a benefit, the improvement was frequently conceptualized not as progress per se but as the preservation of function or prevention of decline. This perspective was valued as meaningful in the context of a degenerative condition, even if changes were not dramatic.

“If I hadn’t done this treatment, I don’t know how I’d be now. Maybe I’d be worse, but I don’t know.” (05, T+GVRE+tDCS)

On the other hand, participants who felt the intervention had not brought any meaningful change typically cited external or pre-existing factors, such as their consistent lifestyle, expectations, or doubts about the intervention’s specificity for Parkinson’s disease, as influencing their experience. For example:

“No, the program didn’t change anything for me. I kept the same routine from start to finish.” (02, T)

### Engagement, motivation, and acceptability

Attendance to the planned sessions remained high, with 92% of all scheduled training days being completed with good compliance. This high level of adherence to the intervention highlights the strong engagement generated by the program, which is further illustrated through participants’ narratives.

A strong sense of commitment and personal responsibility was the most prominent and consistent motivational factor across all groups. Many participants described an intrinsic motivation to follow through on what they had started, grounded in their own values and self-discipline. This motivation was often seen as part of their character and life philosophy. Support from the research team and the structure of the sessions was also seen as an important factor that helped participants stay committed:

“When I start something, I finish it… whatever they assign me, I’ll do it.” (12, T+GVRE+tDCS)

“More than the treatment itself, what stood out was the human interaction. There was no sense of distance like ‘I’m here and you’re there.’ The atmosphere was relaxed, and at no point did I feel uncomfortable.” (06, T+GVRE+tDCS)

The addition of gamification and technological elements were a novelty component that boosted engagement for both groups including the GVRE. Following this line of reasoning, some participants in the Treadmill group claimed it was a boring and non-stimulating experience for them.

“What kept me motivated was the expectation of discovering something new each day, what we would do, what I’d see, the landscape, the challenge.” (07, T+GVRE)

“Just doing the treadmill, just walking, without anything else. It was not very stimulating, I think. Well, that’s the impression I have.” (15, T)

Fatigue was acknowledged by some participants, especially in the Treadmill group and as the program intensified, as a barrier for their adherence to the intervention. Other comorbidities that were also present arose in some of the participants’ discourse, pointing them out as limiting factors within the intervention, or even as a potential driver of flare-ups.

“At the beginning, the first week was perfect… But by week seven, I couldn’t go on. By then, it was just fatigue I was feeling… The last few minutes felt endless.” (02, T)

“The respiratory problem limits me a lot. I have other ailments like bronchitis and so on, so I don’t have much choice.” (10, T)

Overall, integration showed discrepancy/expansion for gait and dual-task domains: mean gait speed and dual-task costs were largely stable, yet participants reported meaningful gains in confidence, self-monitoring, and context-specific mobility strategies. Clinical outcomes showed confirm/expand: modest balance improvements aligned with perceived functional stability and maintenance. Engagement outcomes also showed confirm/expand, with qualitative accounts clarifying mechanisms underlying high adherence (novelty, supervision, supportive atmosphere).

## Discussion

This preliminary mixed-methods RCT evaluated a 6-week treadmill-based gait training program enriched with a GVRE and tDCS in people with mild-to-moderate PD. Using a mixed-methods explanatory sequential design, quantitative outcomes were analyzed first, and post-intervention interviews were then used to explain, contextualize, and elaborate the quantitative patterns observed across outcomes (Table 2). The main finding is that the intervention was feasible, well tolerated, and acceptable, with high adherence and no reported adverse events. However, the study did not demonstrate clinically meaningful improvements in the primary gait-speed outcome. The relatively preserved baseline function of the sample should be considered when interpreting the limited objective changes. Most participants had mild-to-moderate disease severity, preserved global cognition, and moderate-to-high physical activity levels, which may have introduced ceiling effects on relevant outcomes. Secondary gait and cognitive findings were small, timepoint-specific, and exploratory, while qualitative data provided contextual insights into participants’ perceived confidence, self-monitoring, walking strategies, and motivation.

### Gait Outcomes: Objective Measures and Subjective Experiences

Because gait speed did not improve significantly and estimated changes remained below the MCID, the present findings do not support a clinically meaningful effect of the intervention on the primary gait endpoint. Changes observed in cadence and stride length–cadence relationships were derived from secondary analyses, were small and timepoint-specific, and should therefore be interpreted as exploratory signals rather than evidence of improved gait performance. These findings may help generate hypotheses about gait regulation strategies, but they require confirmation in larger trials with prespecified biomechanical outcomes. The only exception in performance was a modest yet statistically significant reduction in cadence during the DTM at T1 in the Treadmill+GVRE+tDCS group, which did not persist at T2.

PwPD often exhibit reduced stride length and may increase cadence as a compensatory strategy to maintain walking speed [41]. Previous studies have shown that cadence and other spatiotemporal parameters may differ between overground and treadmill walking in PD, and that the effects of treadmill training on these parameters remain heterogeneous [42–44]. In the present study, the reduction in cadence observed during the motor dual-task condition in the Treadmill+GVRE+tDCS group was small, timepoint-specific, and not accompanied by a clinically meaningful improvement in gait speed. Therefore, this finding should not be interpreted as evidence of improved gait efficiency or normalization of gait, but rather as an exploratory signal that may reflect changes in gait regulation strategy or task prioritization.

Similarly, the observed changes in the stride length–cadence relationship should be interpreted cautiously. The increase in slope in the Treadmill+GVRE+tDCS group at T1 may suggest a possible change in how stride length was scaled relative to cadence, whereas the concurrent reduction in the intercept may indicate a more conservative stepping pattern at a given cadence. At T2, the increase in intercept observed in the Treadmill+GVRE group may suggest longer strides for a comparable cadence [45, 46]. However, these findings were derived from secondary analyses, occurred in selected groups and timepoints, and were not accompanied by meaningful changes in the primary gait-speed outcome. They should therefore be considered hypothesis-generating and require confirmation in larger studies with prespecified biomechanical outcomes.

The qualitative findings provide additional context but should also be interpreted within these limitations. Several participants reported perceived improvements in stride regulation, walking confidence, and walking strategies, whereas others reported little or no change. These participant-reported experiences may reflect increased self-monitoring or perceived control during walking, but they do not constitute objective evidence of functional gait improvement. Overall, the discrepancy between stable objective gait outcomes and perceived changes in mobility highlights the value of integrating quantitative gait assessment with qualitative data.

### Cognitive-motor integration

Although the interventions incorporated cognitive demands, particularly in the GVRE and tDCS conditions, no significant improvements were observed in dual-task gait performance, which remained consistently impaired across groups. This finding stands in contrast to recent evidence suggesting that tDCS may enhance dual-task performance in PD [47]. However, given the preliminary nature of the current study and the limited sample size, these results should be interpreted with caution and warrant further investigation in larger, more powered trials. This lack of consistency was also reflected in the cognitive outcomes. Stroop task performance did not show a consistent pattern of improvement, and the temporary increase in word completion time observed at T1 in the Treadmill+GVRE group was not maintained at follow-up. This isolated finding may reflect random variation, fatigue, task familiarity, or other transient factors rather than a meaningful intervention effect.

Conversely, Go/No-Go accuracy, a proxy for inhibitory control, improved significantly at T2 in the Treadmill+GVRE+tDCS group. This delayed effect may reflect enhanced cognitive control capabilities facilitated by tDCS, which might require longer to consolidate; a pattern that has been observed in previous studies [48]. Notably, this improvement occurred despite the absence of clear gains in gait under cognitive load, matching previous research suggesting that cognitive and motor pathways in PD may respond differently to neuromodulation [49]. However, this delayed inhibitory-control signal is exploratory and should be interpreted considering sample size, the lack of a sham stimulation group and context.

Qualitative accounts suggested an increase of cognitive awareness, caution, and intention in walking. Participants described monitoring the ground more carefully and adjusting stride proactively when facing environmental challenges. These reports may indicate increased self-monitoring or strategy use, but they should be interpreted as participant-perceived experiences rather than objective evidence of improved dual-task gait performance.The lack of improvement in multitasking performance in some individuals may also reflect ceiling effects in already active participants or indicate that short-term interventions may improve strategy before improving output.

### Clinical Outcomes

While quantitative gait outcomes remained largely stable, clinical motor assessments told a more nuanced story. Both the Treadmill+GVRE and Treadmill+GVRE+tDCS groups showed improvements on the UPDRS-III scale at T1 that approached or exceeded the MCID of 5 points [50], though these changes did not reach statistical significance. Only the Treadmill+GVRE group maintained its gain at T2, whereas the Treadmill+GVRE+tDCS group exhibited a significant decline, suggesting that the initial benefits of the combined intervention may not be durable without sustained stimulation or training. In contrast, the Treadmill-only group showed a gradual but significant improvement by T2, reinforcing the idea that even conventional aerobic training can provide meaningful long-term motor benefits in PD [51].

Balance, as measured by the MiniBESTest, improved significantly across all groups, with gains maintained at T2. The mean increase of + 1.48 points was statistically significant but modest and remained below commonly reported thresholds for clinically meaningful change on the MiniBESTest [52]. However, the high level of physical activity observed in the sample, along with the low disease severity, may explain why only a modest improvement was detected. It is possible that participants were already functioning near their ceiling in terms of dynamic balance, thus limiting the measurable impact of the intervention despite potential functional gains.

Although cognitive testing did not explicitly feature in participant narratives, their descriptions of vigilant ground monitoring, cautious stride regulation, and prolonged feelings of ‘being in very good shape’ align with recognized self-management processes in PD, particularly self-monitoring and corrective movement strategies, identified as critical for maintaining functional mobility and independence [53], but the present study cannot determine whether such strategies translated into measurable changes in real-world mobility or cognitive-motor performance.

### Motivation and adherence to intervention

A central theme across interventions was a strong internalized sense of responsibility. Many participants emphasized a personal ethic of completing what they had started, often framed as a core trait or lifelong value. This reflects prior findings suggesting that intrinsic motivation and self-regulation are key predictors of adherence to physical activity programs in older adults and people with chronic conditions [54]. In the context of PD, this sense of obligation was not only self-directed but also relational, grounded in the desire to support scientific progress and not let others down. These motivational narratives align with the Self-Determination Theory [13], which holds that autonomy, competence, and relatedness drive sustained engagement.

A second key motivator, especially relevant in the VR and tDCS groups, was the pursuit of challenge and performance-based self-validation. Participants described deriving satisfaction from overcoming obstacles, earning points, or outperforming previous sessions, experiences consistent with task-related competence and gamification literature [55]. This was evident in the emotional responses to performance variation, which enhanced both engagement and self-monitoring. Such features may be particularly important in neurorehabilitation, where motivation often fluctuates and fatigue or frustration can impede progress [56]. Notably, even participants in the non-gamified Treadmill group found intrinsic motivation in personal endurance and limits-testing, suggesting that tailored challenge, whether cognitive or physical, may serve as a universal lever for engagement [13].

The immersive and dynamic nature of the VR component appeared to be an important source of motivation, cited as breaking the monotony of conventional treadmill walking. This aligns with previous findings that novelty and environmental variability enhance adherence to exercise among people with PD [57, 58]. VR elements appeared to provide not only distraction from physical exertion but also cognitive stimulation, potentially enhancing attentional engagement and masking fatigue, as seen by the diverse experiences detailed by participants. VR was also described as a way to make longer sessions more tolerable, pointing to its potential role in promoting adherence in future home-based or long-duration rehabilitation formats [59].

Despite predominantly positive accounts, participants also described moments of low motivation. Barriers included physical fatigue, concurrent health conditions, fear of falling, poor performance days, and environmental discomfort. These experiences are consistent with known challenges in PD physical activity adherence and reinforce the need to design interventions that not only activate motivation but also support it during dips [60]. In several cases, social reinforcement, whether from family or the research team, was cited as a critical factor in maintaining participation, highlighting the value of relational accountability and the emotional adherence that can accompany well-supported clinical research environments [61].

Consistent with the explanatory sequential design, the qualitative findings expanded the interpretation of the quantitative results by illustrating how participants perceived changes in confidence, self-monitoring, and everyday walking strategies despite limited objective changes in conventional gait outcomes (Table 2). These reports are clinically relevant for understanding acceptability, perceived usefulness, and possible mechanisms of engagement; however, they should not be interpreted as objective evidence of functional improvement. Rather, they generate hypotheses for future studies incorporating real-world mobility outcomes, longer follow-up, and recruitment of participants with greater baseline gait impairment.

### Strengths and Limitations

This study presents several notable strengths that enhance its contribution to PD rehabilitation research. First among them is its explanatory sequential mixed-methods design, which integrated quantitative and qualitative findings to provide a multidimensional perspective on intervention impact. This approach allowed the detection of subjective benefits, such as increased gait confidence and strategic self-regulation, that would have been overlooked in a purely quantitative analysis. The inclusion of a GVRE within treadmill-based training represents another strength, as it introduced cognitively demanding and ecologically valid walking tasks. This is particularly relevant given the real-world dual-task challenges faced by PwPD [4, 9].

High attendance and compliance rates and positive reports of motivation across all groups underscore the intervention’s feasibility and acceptability, which are crucial in designing scalable and sustainable rehabilitation programs [56]. The gamification elements in the GVRE likely played a significant role in improving engagement and promoting sustained effort, consistent with literature on motivation-enhancing technologies in older and neurologically impaired populations [14, 55]. The inclusion of cognitive and kinematic assessments alongside clinical measures and participant narratives allowed for a comprehensive understanding of how motor and cognitive-motor processes responded to the intervention.

However, several limitations must be acknowledged when interpreting this study’s findings. First, the small sample size, obtained due to the preliminary nature of this study (*n* = 23) significantly limited statistical power, especially for detecting group-by-time interactions and changes in more variable outcomes such as executive function and DT performance. Second, the absence of a sham tDCS condition limits attribution of observed effects to neuromodulation and may have introduced expectation or performance bias, particularly for subjective outcomes and qualitative reports. Finally, the sample was characterized by relatively high baseline physical activity and mild-to-moderate disease severity. These features likely introduced ceiling effects in gait speed, balance, and cognitive scores, thereby limiting the ability to detect clinically meaningful improvements; an issue previously observed in similar interventions targeting high-functioning individuals with PD [62]. Supporting this claim, subjective feedback from interviews suggested that participants who were already physically active or functionally stable perceived little change, while those with lower baseline activity, or even sedentary individuals who may be recruited in the future, were more likely to report benefits. This highlights the importance of stratified or tailored approaches in future research.

The intervention’s relatively short duration (six weeks) may also have been insufficient to generate durable changes in neuroplasticity or motor learning. Although some early improvements were noted, such as in stride-cadence scaling and Go/No-Go accuracy, many participants themselves expressed uncertainty about whether the timeframe allowed for meaningful change. Furthermore, while qualitative interviews provided valuable information into participants’ lived experiences, the absence of objective post-intervention assessments in community mobility or real-world DT performance limits conclusions about the ecological validity of this intervention. Future studies could benefit from incorporating wearable technology or ecological momentary assessment to track real-life behavior, outside from clinical and research contexts [63].

## Conclusions

This preliminary mixed methods RCT suggests that a 6-week treadmill program, enriched with a GVRE and tDCS, was safe, feasible, and well accepted in people with mild-to-moderate PD. However, objective gait improvements were limited, and gait-speed changes remained below clinically meaningful thresholds. Exploratory secondary findings and participant-reported experiences suggest possible effects on motivation, confidence, self-monitoring, and cognitive-motor strategy use, but these findings should be considered hypothesis-generating. The high baseline functioning of the sample may have limited measurable change, highlighting the importance of including a higher percentage of more impaired individuals in future trials. Mixed outcomes across measures underscore the value of combining subjective and objective assessments and tailoring interventions to individual profiles. Future larger, adequately powered, sham-controlled trials should examine whether longer and more targeted interventions can produce clinically meaningful improvements, particularly in individuals with greater gait impairment or lower baseline physical activity.

## Supplementary Information

Below is the link to the electronic supplementary material.

Supplementary Material 1 (DOCX 16.7 KB)

## Declarations

### Trial Registration

NCT05243394
