# Supplementary material for: A treadmill training program in a gamified virtual reality environment combined with transcranial direct current stimulation in Parkinson’s Disease: Preliminary results of a mixed methods randomized controlled trial
Source: Neurol Sci. 2026 Jun 12;47(7):564. doi: 10.1007/s10072-026-09136-8 (PMC13260263; doi:10.1007/s10072-026-09136-8)
Supplement: Supplementary file 1 — Supplementary Material 1 (DOCX 16.7 KB) [file 10072_2026_9136_MOESM1_ESM.docx]

| Supplementary Table S1. Description of the treadmill, GVRE, and tDCS intervention components | |
| --- | --- |
| **Component** | **Description** |
| Intervention arms | Participants were allocated to one of three intervention arms: Treadmill, Treadmill+GVRE, or Treadmill+GVRE+tDCS. The treadmill training structure was common to all groups. The GVRE was added in the two GVRE arms, and anodal tDCS was added only in the Treadmill+GVRE+tDCS arm. |
| Intervention duration and frequency | The intervention lasted 6 weeks, with 2 sessions per week, for a total of 12 sessions. |
| General treadmill structure | All sessions began with a 3-minute warm-up block of treadmill walking without GVRE. Active walking duration progressed from 20 minutes in week 1 to 45 minutes in week 6. |
| Block structure and rest periods | Week 1 consisted of 4 blocks of 5 minutes. Week 2 consisted of 5 blocks of 5 minutes. Weeks 3–6 consisted of 5 blocks of increasing duration: 6, 7, 8, and 9 minutes, respectively. A 3-minute rest period was provided after each block. |
| Treadmill speed progression | In week 1, treadmill speed was set at 85% of the participant’s normal overground walking speed, determined from the 10-meter walking test. Week 2 was set at 90%. From weeks 3 to 6, speed increased by 5% each week, corresponding to approximately 95%, 100%, 105%, and 110% of baseline walking speed. |
| GVRE equipment and set-up | In the GVRE arms, a wide-screen TV was positioned in front of the treadmill. Two HTC SteamVR 2.0 sensors collected information from two HTC Vive 3.0 trackers strapped to the participant’s feet. The movement data were processed by a computer and represented in the virtual environment on the screen. |
| Participant interaction with the GVRE | Participants interacted with the virtual environment by modifying their walking pattern on the treadmill. Foot-tracker data allowed the system to accurately represent feet movement and obstacle negotiation within the virtual scenario. |
| GVRE setting and narrative task | Three virtual environments were used: park, city, and countryside. Each environment was used for 2 weeks. The GVRE was structured around a “walking your dog” task. Participants selected the dog’s color and name, and the dog walked beside them throughout the sessions. The dog animation progressed across the intervention: slow walking in weeks 1–2, faster walking in weeks 3–4, and light trotting in weeks 5–6, reinforcing the perception of progression. |
| Gamification elements and progression | Gamification elements included a progress bar, green/yellow/red performance indicators for obstacles, progressive difficulty, customized level progression, scoring systems, visual reward metrics, and obstacle-management scores. Difficulty was progressed through five mechanisms: treadmill speed, obstacle frequency, distractors, visibility, and path width. Obstacle frequency progressed from every 30 seconds in weeks 1–2, to every 25 seconds in weeks 3–4, and every 20 seconds in weeks 5–6. Distractors appeared on weeks 2, 4 and 6. Visibility was limited through the change of environmental light and the use of fog. Path width was progressively narrowed, going from 4m in weeks 1-2, to 3m in weeks 3-4, to 2m in weeks 5-6. |
| Level progression | Participants could increase one level after the first session if they completed 3 blocks with more than 80% success in obstacle management. Level promotion was limited to one level per week. |
| tDCS component | In the Treadmill+GVRE+tDCS arm, anodal tDCS was applied concurrently with GVRE treadmill training as an adjunct intended to modulate cognitive-motor control processes during task-oriented gait practice. Two saline-soaked sponge electrodes with a total surface of 35 cm² were placed on the scalp and secured with straps. The anode was placed over the left dorsolateral prefrontal cortex, identified as F3 according to the international 10–20 EEG system. The cathode was placed over the right dorsolateral prefrontal cortex. After the 3-minute warm-up block, stimulation was turned on. Anodal tDCS was delivered for 20 minutes at 2 mA, reached after a 30-second ramp-up. Stimulation was not stopped during walking breaks. |
